# Morphological, Physiological, and Transcriptional Changes in *Crocus sativus* L. Under In Vitro Polyethylene Glycol-Induced Water Stress

**DOI:** 10.3390/biology14010078

**Published:** 2025-01-15

**Authors:** Suman Gusain, Rohit Joshi

**Affiliations:** 1 Division of Biotechnology, CSIR-Institute of Himalayan Bioresource Technology, Palampur 176061, Himachal Pradesh, India; suman6gusain@gmail.com; 2 Academy of Scientific and Innovative Research (AcSIR), Ghaziabad 201002, India

**Keywords:** antioxidant activity, drought stress, morpho-physiological analysis, saffron, transcript analysis

## Abstract

Saffron (*Crocus sativus* L.), a perennial geophyte from the Iridaceae family, blooms in autumn and thrives in Mediterranean-like climates. It is highly valued for its therapeutic and commercial uses. While saffron cultivation generally requires minimal water, insufficient irrigation can negatively impact its yield. Although numerous studies have explored the detrimental impact of drought on saffron under field conditions, its impact in vitro remains largely unexplored. The present study aims to investigate the effects of polyethylene glycol (PEG) 6000 at concentrations of 0%, 5%, and 10% in inducing drought stress on saffron shoots under controlled conditions. The research focuses on evaluating morphological, physiological, and biochemical changes and analyzing the expression of drought-responsive genes. Shoot establishment was carried out on Murashige and Skoog (MS) medium supplemented with 6 mg/L 6-benzyladenine (BAP) and 1 mg/L naphthaleneacetic acid (NAA), while PEG 6000 was used to induce drought stress. Various morphological, biochemical, and molecular parameters were assessed 30 days after stress induction. Increasing PEG concentrations in the medium significantly reduced shoot regeneration, leading to increased apical tissue browning. Significant chlorophyll and carotenoid level changes were observed in shoots exposed to higher PEG concentrations. PEG-induced drought led to decreased plant growth and biomass and lowered relative water content of leaves. Lipid peroxidation, membrane damage, and H_2_O_2_ content increased, indicating heightened stress levels. Proline concentration significantly increased in plants subjected to 5% and 10% PEG compared to controls. Non-enzymatic antioxidant activity (phenolics, flavonoids, % inhibition, total reducing power, and total antioxidant activity) also increased with the severity of stress. In contrast, a decrease in the activity of superoxide dismutase (SOD) and peroxidase was observed in PEG-treated shoots. Significant changes in the expression of drought-related genes, such as *DREB1*, *DREB2*, *AREB1*, *DHN1* (*Dehydrin*), and *SnRK2*, were observed in shoots exposed to 5% and 10% PEG. In conclusion, the study highlights that PEG, as an inducer of drought stress, negatively impacts saffron’s growth and physiological responses under in vitro conditions. It also triggers significant changes in biochemical and molecular mechanisms, indicating the plant’s susceptibility to water scarcity.

## Simple Summary

Environmental fluctuations are expected to cause climatic extremes, such as prolonged droughts, impacting the productivity of plants adapted to Mediterranean climates. Saffron, an important spice known for its low water requirements, faces significant threats to yield productivity due to water scarcity. This study evaluated the effects of polyethylene glycol (PEG) 6000-induced drought stress (0%, 5%, 10%) on *in vitro* saffron shoots cultured on MS media supplemented with BAP and NAA. Higher PEG levels reduced shoot regeneration, increased apical browning, and altered chlorophyll and carotenoid levels. Drought stress also decreased growth, leaf water content, and enzymatic antioxidant activity (SOD and peroxidase) while increasing lipid peroxidation, membrane damage, proline accumulation, non-enzymatic antioxidant activity, and transcript abundance of drought-related genes. These findings highlight saffron’s susceptibility to drought, even under in vitro conditions, and underscore the importance of developing drought-resistant varieties.

## 1. Introduction

Water is a critical component of plant physiology, constituting approximately 80 to 95% of a plant’s fresh biomass [1]. It plays a vital role in facilitating the transport of essential nutrients and minerals from the soil to the plant, which is fundamental for growth and development. A water deficit disrupts these processes, leading to impaired metabolic functions and significant reductions in yield. In vitro plant culture provides a controlled environment to study stress tolerance mechanisms by regulating light intensity, temperature, nutrient availability, and stress factors [2]. This approach is beneficial for understanding plant responses to drought stress. Research has consistently shown that enhanced antioxidant activity [3] and altered expression of various genes such as *AREB*, *DREB*, *DHN*, and *SnRK* play a significant role in regulating plant responses to drought. These responses include the accumulation of osmoprotectants and scavenging reactive oxygen species (ROS), mediated through both ABA (abscisic acid)-dependent and independent pathways. Gaining insights into these drought stress responses is essential for developing plant varieties capable of thriving in extreme environmental conditions, ultimately improving agricultural productivity in water-limited regions.

*Crocus sativus* L. (saffron) is an economically significant plant indigenous to the Mediterranean region and is now predominantly cultivated in Iran, India, Greece, Morocco, and Spain. Environmental factors such as photoperiod, temperature, and precipitation are significant determinants affecting saffron growth and development. While saffron is suited to temperate, semi-arid, and arid climates and requires less water, recent reports indicate adverse effects of water stress on saffron [4,5,6]. Drought stress in saffron reduces relative water content (RWC), decreases leaf length, and produces fewer leaves [7]. Additionally, studies have shown alterations in biomass, reductions in photosynthetic pigments, changes in secondary metabolites, and variations in antioxidant enzyme activity under drought stress [8,9]. While the effects of drought stress on saffron’s physiological and biochemical traits in field conditions have been explored, its impact on gene expression remains poorly understood. To address this gap, this study evaluates the effects of polyethylene glycol (PEG) 6000-mediated drought stress on saffron under in vitro conditions. PEG 6000 is commonly used as a drought-inducing agent due to its non-toxic and non-penetrating properties, effectively simulating water-deficit conditions. Although in vitro studies do not fully replicate field conditions, they provide a unique opportunity to study plant responses in a controlled environment, minimizing external variables. This approach allows for a detailed investigation of physiological and biochemical responses to drought stress.

In addition to morphological and physiological assessments, this study aims to explore the regulation of drought-responsive genes, such as *DREB1*, *DREB2*, *AREB1*, *SnRK2*, and *DHN1*, which play crucial roles in stress signaling and adaptation in plants. By examining the expression of these genes, the research seeks to uncover the molecular mechanisms underlying saffron’s response to drought. This study addresses a critical knowledge gap by investigating the impact of drought stress on in vitro saffronproliferation, focusing on biochemical, physiological, and gene expression changes that regulate stress-related traits. The findings will enhance our understanding of saffron’s mechanisms for coping with drought stress and provide a foundation for future research to improve its resilience in water-scarce environments.

## 2. Materials and Methods

The current experiment was performed in the Plant Tissue Culture Laboratory, Department of Biotechnology, CSIR-IHBT, Palampur, H.P. Corms were used as explants for establishing saffron shoot cultures.

### 2.1. Plant Material and Growth Conditions

Corm tunics were manually peeled, and the corms were rinsed under running tap water for an hour. Residual tunics around the buds and damaged parts were excised using a sterile blade. For surface sterilization, the corms were cleaned using a sable hair brush and Tween-20, rinsed with distilled water, and treated with 0.4% Bavistin (*w*/*v*) and 0.4% (*w*/*v*) antibacterial solutions for 30 min, followed by three to four rinses with distilled water. Further surface sterilization was conducted in a laminar flow cabinet. Corms were treated with 70% ethanol for 1 min and then with 0.1% (*v*/*v*) HgCl_2_ (mercuric chloride) for 15 min. Afterward, explants were washed with autoclaved distilled water to remove sterilant residues [10].

For bud induction, corms were cultured in MS basal medium [11] containing 30 g/L sucrose and 8 g/L agar. The pH of the media was adjusted to 5.75–5.80 ± 0.2 before autoclaving at 121 °C for 20 min. After 30 days, the corms were sub-cultured in MS medium comprising 6 mg/L BAP and 3% sucrose to promote bud elongation [10]. Cultures were maintained at 15 °C under aseptic conditions.

For shoot multiplication, germinated buds were shifted to MS medium with various concentrations of meta-topolin (0.25, 0.50, 0.75, 1 mg/L), 6-Benzylaminopurine (BAP 1, 2, 3, 4, 5, and 6 mg/L), and Naphthalene acetic acid (NAA 0.2, 0.4, 0.6, 0.8, and 1 mg/L), either alone or in combination (Table 1). After 45 days, the multiple shoot cultures were evaluated based on morphological characteristics. The optimized medium and explants derived from *in vitro* cultures were subsequently utilized for water stress experiments.

### 2.2. Induction of Drought Stress

In vitro-raised multiple shoot clumps were cultured in the suitable shoot multiplication medium (M11) containing 6 mg/L BAP and 1 mg/L NAA, complemented with different concentrations of PEG 6000 (0%, 5%, 10%) to stimulate drought stress. Three explants were placed into each 250 mL flask in replicates of five. Cultures were maintained at 15 °C under aseptic conditions for 30 days. All morphological, biochemical, and molecular traits were analyzed after the completion of the experiment.

### 2.3. Analysis of Plant Biomass

For biomass analysis, plant samples were rinsed with distilled water and blot-dried, and then the fresh weight (FW) of the plants was determined using an electronic weighing balance (Igene Labserve, New Delhi, India). The plant samples were dried in a hot air oven until completely dried. The dry matter content of the samples was quantified to determine the final dry weight (DW).

### 2.4. Assessment of RWC

The RWC of leaves was determined using the Barrs and Weatherly method [12]. Initially, the FW of the leaf samples was obtained using an electronic weighing balance. The leaves underwent a 24-h incubation in tubes filled with distilled water. After 24 h, the samples were removed from the tubes, excess water from the leaf surface was blot-dried, and the turgid weight (TW) was determined. These samples were then dehydrated in an oven maintained at 60 °C to determine the DW of the samples. RWC was calculated using the following formula:

RWC% = (FW − DW)/(TW − DW) × 100

### 2.5. Electrolyte Leakage

To determine electrolyte leakage, 200 mg of leaf samples were placed in a tube filled with 20 mL of deionized water. The tubes were set in an incubator shaker for 24 h at normal temperature, after which the electrical conductivity (E1) of the water was measured using an EI Deluxe conductivity meter (Electronics India, Haryana, India). After determining E1, the tubes were heated at 121 °C for 30 min, and E2 was assessed after the samples were allowed to cool for 30 min at room temperature (RT).

Electrolyte leakage % = E1/E2 × 100

### 2.6. Measurement of Chlorophylls, Carotenoids, and Chlorophyll Fluorescence

The chlorophyll and carotenoid measurements in plant samples were conducted using the Arnon method [13]. This method involves solvent-dependent pigment extraction, followed by absorbance measurement using a spectrophotometer. A sample of 100 mg leaf tissue was homogenized with 80% acetone and centrifuged at 8000 rpm for 20 min (Sigma 3-16 KL, Osterode am Harz, Germany). The upper phase was aliquoted into a new tube, and the process was continued until the supernatant became clear. The absorbance was recorded at 470 nm, 663 nm, and 645 nm using a spectrophotometer (Multiskan Skyhigh, Thermo Fisher Scientific, Cleveland, OH, USA). The concentration of chlorophyll and carotenoids was determined using the following equations:

Chl a—(12.7 × A663 − 2.69 × A645) × V × W/1000 Chl b—(22.9 × A645 − 4.68 × A663) × V × W/1000 Carotenoids—1000 × A470 − 3.29 × Chl a − (104 × Chl b)/198 V = volume of extracted solution in mL, W = weight of fresh sample (g)

To acquire fluorescence measurements, the leaves were dark-treated for thirty minutes. This dark adaptation was crucial for measuring the potential quantum efficiency of PSII, represented as Fv/Fm (variable fluorescence/maximum fluorescence). This value is critical for assessing photosynthetic performance, indicating energy conversion efficiency into chemical energy during plant photosynthesis. Following the dark adaptation, the Fv/Fm values were measured in accordance with the manufacturer’s instructions using the FluorCam system (Photon Systems Instruments, Brno, Czech Republic).

### 2.7. Proline Estimation

To determine free proline content, we followed the experimental approach of Bates et al. [14]. The method includes extracting 300 mg of plant leaf sample with 3 mL of 3% solution of sulfosalicylic acid. The mixture was subjected to centrifugation for 20 min at 3000 rpm. Next, 1 mL of the upper aqueous phase was pooled with 1 mL of 2.5% acid ninhydrin and 1 mL of 60% anhydrous acetic acid. The resulting mixture was heated for an hour in a water bath maintained at 100 °C. The samples were extracted with 2 mL of toluene after 30 min in an ice bath. The absorbance was recorded at 520 nm after 30 min.

### 2.8. H_2_O_2_ and Lipid Peroxidation Estimation

Leaf sample of 100 mg homogenized with 0.1% TCA (Trichloroacetic acid) was spun down at 10,000 rpm for 20 min at 4 °C. The aqueous phase after centrifugation was used as an extract to estimate H_2_O_2_ and MDA (malondialdehyde). The H_2_O_2_ level was estimated using the method of Sergiev et al. [15]. A 0.5 mL sample solution was reacted with 0.5 mL of 10 mM phosphate buffer (pH 7.0) and 1 M potassium iodide. Absorbance was taken at 390 nm (Multiskan Skyhigh, Thermo Fisher Scientific, Cleveland, OH, USA).

Lipid peroxidation was estimated by quantifying the MDA content in the sample extract, and 0.25 mL of extract was combined with 1 mL of a 20% solution of TCA with 0.5% TBA (Thiobarbituric acid). The set was heated at 95 °C for 30 min. After that, the samples were spun down at 12,000 g for 10 min at 4 °C. Sample quantification was performed at 450 nm, 532 nm, and 600 nm.

### 2.9. Enzymatic and Non-Enzymatic Antioxidant Activity

A methanolic extract of the dried plant sample was prepared for non-enzymatic antioxidant estimation. For sample extraction, 100 mg of dried sample was combined with 10 mL of 80% methanol. The tubes were left to stand overnight in the dark. The samples were sonicated (Elma S 300 H, Elma company, Germany) for 30 min to facilitate extraction further. The upper phase was pipetted into a fresh tube, and the process was repeated three times. The pooled supernatant was concentrated using a vacuum rotary evaporator at 40 °C. The resulting concentrate was resuspended in 4 mL of 80% methanol and filtered across a 0.2 μm syringe filter. The sample extract was kept at 4 °C for subsequent analysis.

The total phenol was estimated according to Ainsworth and Gillespie [16] using the Folin-Ciocalteu reagent. A 0.1 mL filtrate was combined with 1.2 mL of 10% Folin reagent. The resulting mixture was briefly vortexed to ensure thorough mixing, followed by incubation in the dark for 10 min. Afterward, 0.96 mL of 10% Na_2_CO_3_ (sodium carbonate) was added to the mixture. The resulting mixture was then dark-adapted for another 2 h. Absorbance was taken at 765 nm. Total phenolic content (TPC) was calculated as gallic acid equivalent (GAE).

The total flavonoid was quantified according to Kalita et al. [17], and 0.2 mL filtrate was combined with the reaction solution containing 0.6 mL of methanol, 0.1 mL of aluminum chloride, and 0.1 mL of potassium acetate. To make the final volume of 3 mL, deionized water was added to the solution. The samples were kept in the dark for 30 min. OD (optical density) was taken at 415 nm. Total flavonoid was quantified as mg quercetin equivalent per g DW.

The DPPH (2,2-diphenyl-1-picrylhydrazyl) inhibition activity of the extract was measured following the protocol described by Susanti et al. [18]. A 1.5 mL aliquot of 0.004% DPPH solution was combined with 0.25 mL extract. The mixture was incubated in the dark for 30 min. Absorbance was recorded at 515 nm, and the percentage inhibition was calculated using the following formula:

% Inhibition = [(ADPPH − Asample)/ADPPH] × 100

The ferric-reducing antioxidant capacity (FRAC) of the sample was determined following the method described by Oyaizu [19]. Sample extract of 0.5 mL was combined with 1.5 mL of 0.2 M potassium phosphate buffer and 1.5 mL of 1% potassium hexacyanoferrate (III). The reaction mixture was placed into a water bath maintained at 50 °C for 20 min. After incubation, 1.5 mL of 10% TCA was mixed into the solution and centrifuged at 6000 rpm for 10 min. Following centrifugation, 1.8 mL of deionized water was added to 1.8 mL of the supernatant. Finally, 0.36 mL of 0.1% ferric chloride (FeCl_3_) solution was added to the sample solution. The absorbance of the sample was assessed at 700 nm.

The total antioxidant capacity (TAC) of the sample extract was estimated according to Prieto et al. [20]. Sample extract of 0.5 mL was combined with 4.5 mL of assay solution comprising 0.6 M sulfuric acid, 28 mM sodium phosphate, and 4 mM ammonium molybdate; 0.5 mL of 45% ethanol was used as a substitute for the plant sample extract for the blank. The tubes were heated at 95 °C for 90 min. Following incubation, the reaction was terminated at room temperature. The total antioxidant capacity of the samples was assessed at 695 nm.

Superoxide dismutase (SOD) activity was measured according to Beauchamp and Fridovich [21]. A 500 mg plant sample was homogenized with 3 mL of 0.1 mM phosphate buffer and 2 mL of 0.5 mM EDTA (ethylenediaminetetraacetic acid) and subsequently spun down at 12,000 rpm for 20 min at 4 °C. The resulting upper phase was used for the analysis of enzyme activity. A mixture containing 0.1 mL of the extract, 0.075 mM NBT (nitroblue tetrazolium), 14.5 mM methionine, 0.1 mM EDTA, 50 mM potassium phosphate buffer, and 0.004 mM riboflavin was placed under light for 15 min. A tube without the enzyme was the control, while a non-incubated mixture was the blank. Following incubation, sample absorbance was taken at 560 nm. One enzyme unit was determined to have a 50% reduction in absorbance relative to the control.

Peroxidase (POD) activity was estimated with the method of Chance and Maehly [22]. The reaction mixture (1 mL) contained 0.5 mL of 50 mM phosphate buffer (potassium-based, pH 7.0), 0.3 mL of 20 mM pyrogallol, and 0.1 mL of sample extract. The reaction was triggered by adding 0.1 mL of 0.5% (*v*/*v*) hydrogen peroxide to the mixture. A reaction mixture without extract was used as the blank. The linear increase in absorbance was recorded after intervals of 30 s for 5 min at 420 nm using a spectrophotometer (Multiskan Sky High, Thermo Fisher Scientific, Cleveland, OH, USA).

### 2.10. RNA Isolation and PCR Evaluation

RNA was isolated from leaf samples using the iRIS method, and the purified RNA was quantified using a micro duo plate reader (Multiskan Sky High, Thermo Fisher Scientific, USA). cDNA synthesis for the first strand was carried out using the Verso cDNA Synthesis Kit (Thermo Fisher Scientific, USA) following the manufacturer’s guidelines. For gene expression analysis, contigs of *DREB1*, *DREB2*, *AREB*, *SnRK2*, and *DHN1* were retrieved from the *C. sativus* online transcriptomic database (SRR18777206). Primers were designed using the Primer3 online tool based on the contig sequences. Tubulin was taken as the internal control (Table 2). Gene expression was analyzed using quantitative real-time PCR (BIO RAD CFX Opus 96, Bio-Rad, California, USA). Tubulin and 18s rRNA were used as internal controls.

### 2.11. Data Analysis

The data were evaluated using one-way analysis of variance (ANOVA) with Origin 2024b software. Each treatment comprised five replicates containing three saffron shoot clumps (explants), resulting in 15 explants per treatment. The results represent the mean ± standard error. The Tukey test was used to determine significant differences between the means at *p* ≤ 0.05.

## 3. Results

### 3.1. Establishment of Multiple Shoot Culture

**Figure 1:**
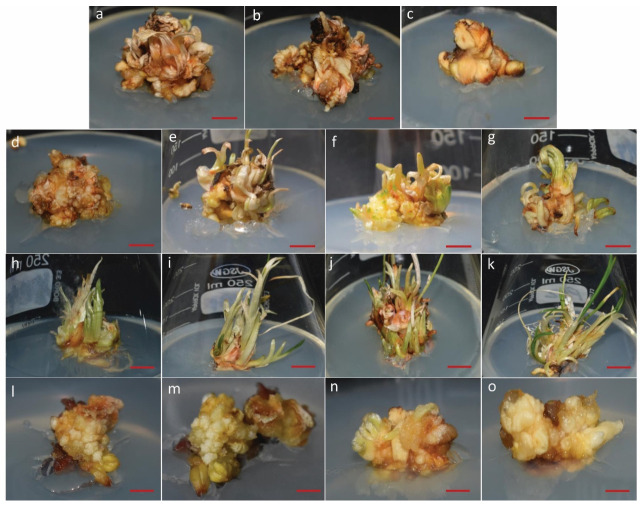
Effect of various concentrations of plant growth regulators on multiple shoot proliferation in *Crocus sativus* L. Cultures were treated with different concentrations of BAP, NAA, and meta-topolin (**a**) Shoot proliferation response with 1 mg/L BAP, (**b**) 2 mg/L BAP, (**c**) 3 mg/L BAP, (**d**) 4 mg/L BAP, (**e**) 5 mg/L BAP, (**f**) 6 mg/L BAP, (**g**) 6 mg/L BAP + 0.2 mg/L NAA, (**h**) 6 mg/L BAP + 0.4 mg/L NAA, (**i**) 6 mg/L BAP + 0.6 mg/L NAA, (**j**) 6 mg/L BAP + 0.8 mg/L NAA, (**k**) 6 mg/L BAP + 1 mg/L NAA, (**l**) 0.25 mg/L meta-topolin, (**m**) 0.50 mg/L meta-topolin, (**n**) 0.75 mg/L meta-topolin, (**o**) 1 mg/L meta-topolin. Scale bar = 1 cm.

The optimized saffron multiple shoot proliferation protocol generated sufficient plant material for in vitro drought stress studies. At minimal concentrations of BAP (1–5 mg/L), delayed growth and browning of shoots were observed, indicating poor development. However, at 6 mg/L BAP, there was a marked improvement, with healthy multiple shoot clumps forming, suggesting that higher BAP concentrations promote shoot multiplication. Adding NAA further enhanced shoot development, with 1 mg/L NAA combined with 6 mg/L BAP (M11) yielding the best shoot and leaf growth results. In contrast, meta-topolin, at the concentrations tested, did not induce any significant response within one month (Figure 1).

### 3.2. Assessing Morphological and Physiological Adaptations to Drought Stress

**Figure 2:**
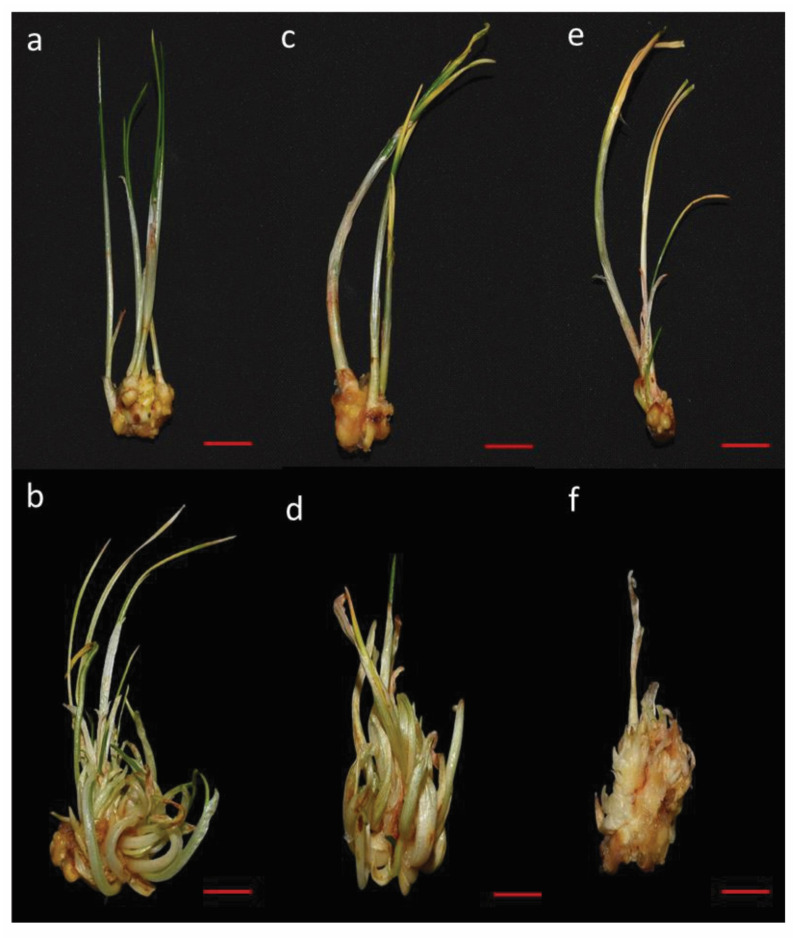
Effect of various levels of drought stress on multiple shoot proliferation of saffron (*Crocus sativus* L.) cultured on MS medium supplemented with 6 mg/L BAP and 1 mg/L NAA (**a**,**b**) Control (0% PEG) (**c**,**d**) 5% PEG (**e**,**f**) 10% PEG. Scale bar = 1 cm.

The varying concentrations of PEG influenced the qualitative properties of in vitro regenerated shoots, such as growth and biomass. Moderate water stress induced by 5% PEG did not significantly affect plant biomass compared to the control. However, plant biomass was significantly reduced under severe water stress (10% PEG) compared to moderate stress (5% PEG) and control conditions. Shoots subjected to 10% PEG exhibited yellowing and browning at the tips (Figure 2).

The total number of shoots decreased as the PEG concentration increased. Explants grown in media with 5% and 10% PEG developed fewer shoots than those in the PEG-free control. The water stress induced by PEG also adversely affected biomass, shoot length, and the FW and DWs of the shoots. The fresh-to-dry weight ratio declined from 10.6 in the control to 9.49 at 5% PEG and further to 5.65 at 10% PEG (Table 3).

**Figure 3:**
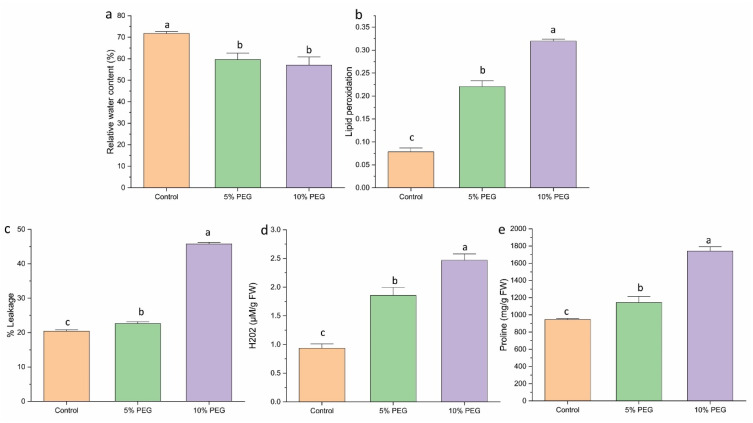
The effect of various concentrations of PEG on (**a**) RWC, (**b**) lipid peroxidation, (**c**) % leakage, (**d**) H_2_O_2_, (**e**) proline content of saffron multiple shoots. The data are the mean ± SE of the three replicates. This means that the same letter does not differ significantly at the 0.05 probability level, according to the Tukey test.

Additionally, the RWC in the leaves declined as the severity of stress increased (Figure 3a). Plants under normal conditions retained a high RWC of 78.43%, whereas those subjected to 5% PEG and 10% PEG showed a reduction in RWC to 66% and 57%, respectively.

### 3.3. Evaluating the Effects on Lipid Peroxidation and Membrane Damage

MDA levels in multiple shoots were measured to evaluate lipid peroxidation under various PEG concentrations (Figure 3b). PEG treatment notably elevated MDA levels in shoots compared to the control. The 5% and 10% PEG treatments resulted in a 3.14 and 4.4-fold increase in MDA accumulation, respectively, relative to the control. The degree of cell membrane leakage induced by PEG showed a positive correlation with MDA content in the tissues (Figure 3c). The maximum leakage was observed in the 10% PEG treatment (45%), followed by a reduction to 22% in the 5% PEG treatment.

### 3.4. H_2_O_2_ Determination

The hydrogen peroxide level significantly increased with the severity of stress. Treatment with 5% and 10% PEG resulted in increased H_2_O_2_ content by 2 and 2.6 times, respectively, relative to the control (Figure 3d). This suggests that PEG-induced stress leads to elevated oxidative stress, as indicated by the higher H_2_O_2_ levels.

### 3.5. Effect of Proline Content

PEG-mediated water deficit resulted in a significant rise in proline levels in shoots. Under 5% PEG treatment, proline content increased by 1.2 times, while 10% PEG treatment resulted in a 1.8-fold increase (Figure 3e). The rise in proline content helps plants maintain osmotic balance during stress conditions.

### 3.6. Effect of PEG on Chlorophyll, Carotenoid, and Chlorophyll Fluorescence

**Figure 4:**
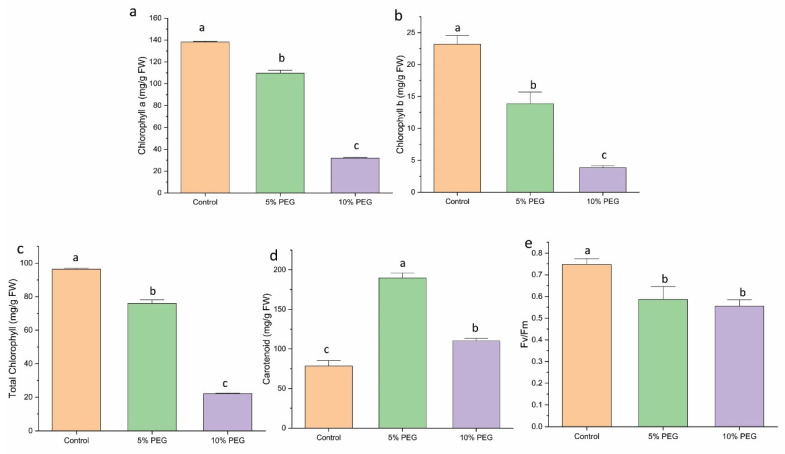
The impact of different concentrations of PEG on (**a**) chlorophyll a, (**b**) chlorophyll b, (**c**) total chlorophyll content, (**d**) carotenoid, and (**e**) Fv/Fm of saffron multiple shoots. The data are expressed as the mean ± standard error (SE) of three replicates. This means that the same letter does not differ significantly at the 0.05 probability level, according to the Tukey test.

Drought stress at both 5% PEG and 10% PEG concentrations caused a comparable and significant decrease in chlorophyll content (a, b, total) in saffron shoots (Figure 4a–c). The carotenoid contents showed a substantial increase under PEG-induced stress. The 5% PEG-treated plants showed a 2.8-fold increase in carotenoid content, while the 10% PEG treatment showed a 1.5-fold increase compared to the control (Figure 4d).

Fv/Fm, an indicator of photooxidative damage to Photosystem II (PSII), decreased with increasing drought stress in plants. Plants without PEG treatment showed minimal to no PSII damage, with Fv/Fm values ranging from 0.7 to 0.8. In contrast, introducing PEG into the growth medium led to a gradual decline in Fv/Fm values. Under 5% PEG, the value decreased by 1.1 times; under 10% PEG, it decreased by 1.3 times, indicating increased stress-induced damage to PSII (Figure 4e).

### 3.7. Effect on Antioxidant Activity

**Figure 5:**
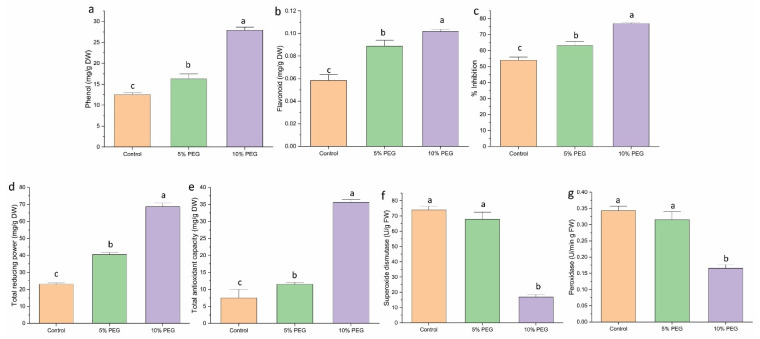
The impact of different concentrations of PEG on (**a**) phenol, (**b**) flavonoid, (**c**) % inhibition, (**d**) total reducing power (TRP), (**e**) total antioxidant capacity, (**f**) SOD activity, and (**g**) peroxidase activity in saffron multiple shoots. The data are presented as the mean ± standard error (SE) of three replicates. This means that the same letter does not differ significantly at the 0.05 probability level, according to the Tukey test.

Non-enzymatic antioxidant activity, such as phenol, flavonoid, FRAC, and TAC, increased under PEG-induced drought stress. Phenol showed a 1.4-fold increase under 5% PEG and a 2.3-fold increase under 10% PEG compared to control (Figure 5a). Flavonoid content increased 1.6 and 2 times under 5% and 10% PEG, respectively (Figure 5b). The reducing power and antioxidant activity also increased by 1.7 and 3 times, 1.5 times, and 5 times under 5% and 10% PEG treatment, respectively (Figure 5c,d). The DPPH % inhibition capacity of the shoots significantly increased by 1.16 and 1.4 times under 5% and 10% PEG treatment, respectively (Figure 5e).

The antioxidant activity of enzymes was measured to evaluate the response of saffron shoots to various concentrations of PEG. The SOD and POD showed an inverse correlation with the intensity of the stress (Figure 5f,g). SOD enzyme activity exhibited a 1.11-fold decrease under 5% PEG and a 4.6-fold decrease under 10% PEG when compared to the control. Similarly, POD activity decreased by 1.0 times under 5% PEG and 2.0 times under 10% PEG compared to the control.

### 3.8. Alteration in Gene Expression Under Drought Stress

**Figure 6:**
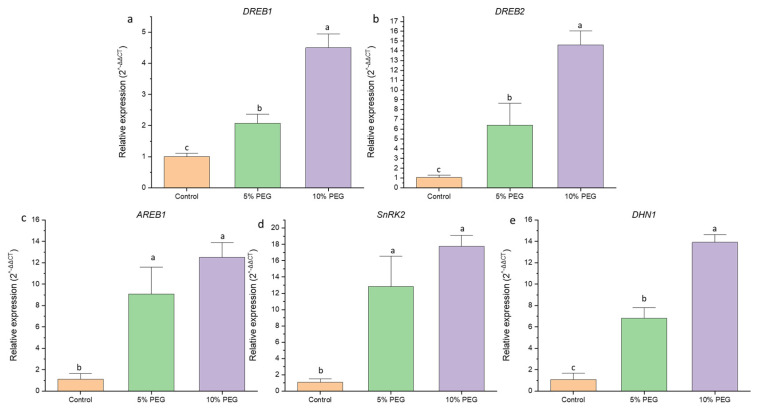
Effect of various levels of PEG6000 (0%—control, 5%, 10%) on the expression of various genes regulating drought stress in saffron. (**a**) on *DREB1* expression, (**b**) on *DREB2* expression, (**c**) on *AREB1* expression, (**d**) on *SnRK2* expression, (**e**) on *DHN1* expression. The data are the mean ± SE of the three biological replicates with *18s RNA* and *tubulin* as reference genes. The same letter does not differ significantly at the 0.05 probability level, according to the Tukey test.

Quantitative real-time PCR analysis revealed an upregulation in gene expression under varying levels of water stress (Figure 6). The expression of *DREB1* increased by 2.1-fold under 5% PEG treatment and 4.5-fold under 10% PEG treatment. Similarly, *DREB2* showed a 6.3-fold increase under 5% PEG and a remarkable 14.6-fold increase under 10% PEG. The expression of *AREB1* also increased significantly, with a 9.0-fold and 12.4-fold rise under 5% and 10% PEG, respectively. Furthermore, *SnRK2* expression exhibited a 12.8-fold increase under 5% PEG and 17.7-fold under 10% PEG, while *DHN1* expression increased by 6.7-fold under 5% PEG and 13.9-fold under 10% PEG compared to the control.

## 4. Discussion

In this study, we highlighted the significant effects of PEG-mediated water stress on the in vitro growth, physiological responses, and stress tolerance mechanisms in *Crocus sativus* L. Optimizing the shoot proliferation protocol was a crucial step in establishing sufficient plant material for drought studies. Our findings indicate that higher BAP concentrations (6 mg/L) significantly enhanced shoot multiplication, suggesting that BAP promotes cytokinin-induced cell division and shoot formation in *C. sativus*. Adding NAA further stimulated shoot and leaf growth, with the 6 mg/L BAP + 1 mg/L NAA combination providing the best results. This combined effect of BAP and NAA coincides with findings in other monocots where cytokinins and auxins have been shown to promote balanced shoot and root formation, enhancing biomass for experimental analyses [23]. Devi et al. [10] observed a comparable effect of BAP and NAA on saffron shoot multiplication, finding that 6 mg/L BAP along with 0.2 mg/L NAA effectively promoted shoot proliferation in saffron.

The application of PEG to simulate drought stress effectively reduced shoot number, length, and biomass, particularly under severe stress conditions (10% PEG). The observed reductions in FW and DW reflect PEG’s impact on osmotic stress, which reduces water availability and cellular turgor, thereby limiting growth. A previous study on *Medicago sativa* supports our findings, indicating that biomass reduction is commonly observed under PEG-induced stress, often associated with inhibited cell expansion and division due to osmotic imbalance [24]. Although moderate stress (5% PEG) had a relatively minor effect on biomass compared to severe stress, it still reduced the fresh-to-dry weight ratio. This reduction suggests that moderate stress alone affects water uptake, causing plants to adapt by adjusting their water content and biomass.

The observed increases in MDA content and electrolyte leakage in PEG-treated plants indicate oxidative stress and membrane damage, both common under water-deficit conditions. Lipid peroxidation, reflected by elevated MDA levels, was significantly higher in shoots under 10% PEG, suggesting that the severity of oxidative damage escalates with increasing drought stress. This is consistent with previous research, where drought-stressed plants exhibit lipid peroxidation due to the generation of ROS such as H_2_O_2_ [25,26]. Electrolyte leakage further confirms membrane destabilization, as leakage was positively correlated with MDA levels, especially under severe stress. Increased H_2_O_2_ levels observed under PEG treatments indicate ROS accumulation, suggesting oxidative stress may be a key factor driving cellular damage in *C. sativus* under drought.

Proline, as a primary compatible solute in higher plants, is essential for regulating energy for growth and survival and detoxifying ROS within cells. Under drought conditions, proline accumulation in plants helps maintain osmotic balance, reflecting an adaptive response commonly observed in plants. In our study, proline accumulation in saffron shoots was significantly higher under 5% and 10% PEG-induced stress compared to the controls.

The decrease in chlorophyll content, particularly in chlorophyll a and b, reveals the adverse impact of drought on photosynthesis. Reduced chlorophyll levels under PEG treatments may be linked to chlorophyll degradation and inhibited synthesis, both of which reduce photosynthetic efficiency. Interestingly, the carotenoid content increased under both levels of PEG stress. A similar observation was recorded in *Pennisetum glaucum*, where drought stress led to a decrease in Chl a and Chl b in IP14599 and IP14222 genotypes compared to control, and an increase in carotenoid level was noted in both genotypes under drought stress [27]. Carotenoids play a protective role by quenching excess light energy and scavenging ROS, thus helping plants mitigate photooxidative stress. The decrease in the Fv/Fm ratio under PEG-induced drought stress suggests increased photoinhibition and potential damage to Photosystem II (PSII), a sensitive indicator of drought-induced photodamage [28,29].

Drought stress led to a substantial increase in non-enzymatic antioxidants, including phenols, flavonoids, and total antioxidant capacity (TAC), critical for scavenging free radicals and preventing oxidative damage to cellular components. This enhancement strengthens the plant’s defense against drought-induced oxidative stress. These findings were consistent with Ahmad et al., who observed increased total phenols, flavonoids, TRP, and TAC in *Stevia rebaudiana* with elevated PEG concentrations in the growth medium [30].

In contrast, enzymatic antioxidants such as SOD and POD showed reduced activity under PEG-induced drought conditions, unlike the increased levels of non-enzymatic antioxidants. This reduction in SOD and POD activity may suggest enzyme inactivation or downregulation under prolonged stress, possibly due to a reallocation of cellular resources toward non-enzymatic antioxidant production as a more efficient protective strategy under severe drought conditions. These findings align with reports by Fan et al. and Bondak et al. that documented decreased SOD and POD activity in cucumber and Egyptian teosinte, respectively, under PEG-induced drought stress [31,32]. Similarly, Zhang et al. observed reduced enzymatic antioxidant activity (SOD and POD) in *Atractylodes lancea* under drought stress [33].

The upregulation of drought-responsive genes, such as *DREB 1* and *2*, *AREB1*, *SnRK2*, and *DHN1*, indicates an active molecular response to water deficit. The expression of DREB and AREB transcription factors suggests the involvement of both ABA-dependent and independent pathways in the stress response, coordinating protective mechanisms such as osmolyte accumulation, ROS scavenging, and cell wall stabilization. In this study, we observed an increase in the expression of *DREB1*, *DREB2*, and *AREB1* under PEG treatment. Consistent with our findings, a study on *H. persicum* also revealed the upregulation of DREB and AREB transcripts under PEG treatment, suggesting a probable role of these transcription factors in regulating the expression of downstream genes responsible for drought tolerance [34]. Furthermore, we observed increased expression of *SnRK2*, a gene linked to cellular water retention and protection against dehydration. Overexpression of *TaSnRK2.9* in tobacco has been shown to facilitate ROS scavenging under drought stress [35]. Similarly, dehydrins are induced by drought stress and play a crucial role in the plant’s response to stress. Collectively, these findings enhance our understanding of the intricate mechanisms underlying drought tolerance in *C. sativus*, paving the way for future research into improving saffron cultivation under water-limited conditions.

## 5. Conclusions

In conclusion, this study demonstrates that *Crocus sativus* can exhibit stress-responsive changes under drought conditions through a combination of morphological adaptations, oxidative stress mitigation, and gene-mediated responses. The insights gained from these observations could enhance breeding programs to improve drought tolerance in saffron and related species, significantly impacting sustainable crop production in arid or water-scarce regions. Future research could further investigate the interaction between growth regulators and PEG stress and the roles of additional antioxidants and drought-responsive genes in enhancing saffron’s resilience to water scarcity.

## Figures and Tables

**Table 1:** Combinatorial effect of plant growth regulators on multiple shoot proliferation in saffron.

Medium	BAP (mg/L)	NAA (mg/L)	Metatopolin (mg/L)	Response After 1 Month
M1	1	-	-	Delayed growth and browning of shoots
M2	2	-	-	Delayed growth and browning of shoots
M3	3	-	-	Delayed growth and browning of shoots
M4	4	-	-	Delayed growth and browning of shoots
M5	5	-	-	Delayed growth and browning of shoots
M6	6	-	-	Healthy multiple shoots
M7	6	0.2	-	Healthy multiple shoots
M8	6	0.4	-	Healthy multiple shoots
M9	6	0.6	-	Healthy multiple shoots
M10	6	0.8	-	Healthy multiple shoots
M11	6	1	-	Healthy multiple shoots with increased leaf length
M12	-	-	0.25	No response
M13	-	-	0.50	No response
M14	-	-	0.75	No response
M15	-	-	1	No response

**Table 2:** List of primers used for expression analysis of drought stress-related genes.

Gene	Sequence	Length (bp)	Product Size	Annealing Temperature (°C)
*AREB1_F*	TTCGACGAGTTCCAGAGCAC	20	87	60
*AREB1_R*	CGTCCACACGTTCCGTAGAA	20		
*DHN1_F*	GGTGGCCACAAGTCGGA	17	50	60
*DHN1_R*	TCTTGTCCGTAGTCGTATCTGT	22		
*DREB1_F*	TCCTCCTACATGACCGTCTC	20	70	60
*DREB1_R*	GGGTCTCGTGGAACTTGGT	19		
*DREB2_F*	CACAATGCCGTCGACAAGAAG	20	79	60
*DREB2_R*	AGCCCTTTCTTGATTTCCGC	20		
*SnRK2_F*	CTACGTGCTCCGTCACCTTT	20	87	60
*SnRK2_R*	TTGACGAGGCACGAGAACAG	20		
*Tubulin_F*	CGTGCGTTTGTTCACTGGTA	20	104	60
*Tubulin_R*	CCCACCTCTTCGTAATCCTTC	21		
*18srRNA_F*	TGTTATTGCCTCAGCCTTCC	20	133	60
*18srRNA_R*	GCGGTTTCTCTGGTTAATTCC	21		

**Table 3:** The impact of various PEG concentrations on the biomass, shoot FW and DW, and FW/DW of in vitro saffron multiple shoots.

	Biomass	Shoot FW (g)	Shoot DW (g)	FW/DW
Control	2.44 ± 0.13 a	2.69 ± 0.15 a	0.25 ± 0.02 a	10.65 ± 0.69 a
5% PEG	2.29 ± 0.05 a	2.57 ± 0.09 a	0.28 ± 0.04 a	9.49 ± 1.2 ab
10% PEG	1.48 ± 0.19 b	1.80 ± 0.19 b	0.32 ± 0.02 a	5.65 ± 0.62 b

Means showing the same letter does not differ significantly at the 0.05 probability level according to the Tukey test.

## Data Availability

Data are contained within the article.

## References

[1] Abbasi T., Abbasi S.A. (2010). Biomass energy and the environmental impacts associated with its production and utilization. Renew. Sustain. Energy Rev..

[2] Bajji M., Lutts S., Kinet J.M. (2000). Physiological changes after exposure to and recovery from polyethylene glycol-induced water deficit in callus cultures issued from durum wheat (*Triticum durum* Desf.) cultivars differing in drought resistance. J. Plant. Physiol..

[3] Mokhtari N., Majidi M.M., Mirlohi A. (2024). Physiological and antioxidant responses of synthetic hexaploid wheat germplasm under drought. BMC. Plant. Biol..

[4] Koocheki A., Karbasi A., Seyyedi M. (2017). Some reasons for saffron yield loss over the last 30 years period. Saffron Agron. Technol..

[5] Sojasi Qidari H., Behrooz Z. (2017). Analysis of the effects of change in cropping pattern due to drought on saffron production in rural areas of the Zebarkhan district villages. Rural Dev. Strateg..

[6] Feroze S.M., Baba S.H., Laitonjam N., Singh R., Thangjam D. (2021). Saffron production depends on rainfall: Empirical evidence from Jammu & Kashmir. SKUAST J. Res..

[7] Maleki M., Ebrahimzade H., Gholami M., Niknam V. (2011). The effect of drought stress and exogenous abscisic acid on growth, protein content and antioxidative enzyme activity in saffron (*Crocus sativus* L.). Afr. J. Biotechnol..

[8] Tavakoli F., Rafieiolhossaini M., Ravash R. (2021). Effects of PEG and Nano-Silica Elicitors on Secondary Metabolites Production in *Crocus sativus* L.. Russ. J. Plant Physiol..

[9] Bistgani Z.E., Barker A.V., Hashemi M. (2024). Physiology of medicinal and aromatic plants under drought stress. Crop J..

[10] Devi K., Sharma M., Singh M., Singh Ahuja P. (2011). In vitro cormlet production and growth evaluation under greenhouse conditions in saffron (*Crocus sativus* L.)—A commercially important crop. Eng. Life Sci..

[11] Murashige T., Skoog F. (1962). A revised medium for rapid growth and bio assays with tobacco tissue cultures. Physiol. Plant.

[12] Barrs H.D., Weatherley P.E. (1962). A re-examination of the relative turgidity technique for estimating water deficits in leaves. Aust. J. Biol. Sci..

[13] Arnon D.I. (1949). Copper enzymes in isolated chloroplasts. Polyphenoloxidase in *Beta vulgaris*. Plant Physiol..

[14] Bates L.S., Waldren R.P.A., Teare I.D. (1973). Rapid determination of free proline for water-stress studies. Plant Soil.

[15] Sergiev I., Alexieva V., Karanov E. (1997). Effect of spermine, atrazine and combination between them on some endogenous protective systems and stress markers in plants. Compt. Rend. Acad. Bulg. Sci..

[16] Ainsworth E.A., Gillespie K.M. (2007). Estimation of total phenolic content and other oxidation substrates in plant tissues using Folin–Ciocalteu reagent. Nat. Protoc..

[17] Kalita P., Tapan B.K., Pal T.K., Kalita R. (2013). Estimation of total flavonoids content (TFC) and antioxidant activities of methanolic whole plant extract of *Biophytum sensitivum* Linn. J. Drug Deliv. Ther..

[18] Susanti D., Sirat H.M., Ahmad F., Ali R.M., Aimi N., Kitajima M. (2007). Antioxidant and cytotoxic flavonoids from the flowers of *Melastoma malabathricum* L.. Food Chem..

[19] Oyaizu M. (1986). Studies on products of browning reactions: Antioxidant activities of products of browning reaction prepared from glucose amine. Jpn. J. Nutr..

[20] Prieto P., Pineda M., Aguilar M. (1999). Spectrophotometric quantitation of antioxidant capacity through the formation of a phosphomolybdenum complex: Specific application to the determination of vitamin E. Anal. Biochem..

[21] Beauchamp C., Fridovich I. (1971). Superoxide dismutase: Improved assays and an assay applicable to acrylamide gels. Anal. Biochem..

[22] Chance B., Maehly A.C. (1955). Assay of catalases and peroxidases. Methods Enzymol..

[23] Pathi K.M., Tula S., Huda K.M.K., Srivastava V.K., Tuteja N. (2013). An efficient and rapid regeneration via multiple shoot induction from mature seed derived embryogenic and organogenic callus of Indian maize (*Zea mays* L.). Plant Signal. Behav..

[24] Zhang C., Shi S., Wang B., Zhao J. (2018). Physiological and biochemical changes in different drought-tolerant alfalfa (*Medicago sativa* L.) varieties under PEG-induced drought stress. Acta Physiol. Plant..

[25] Aghaie P., Tafreshi S.A.H., Ebrahimi M.A., Haerinasab M. (2018). Tolerance evaluation and clustering of fourteen tomato cultivars grown under mild and severe drought conditions. Sci. Hortic..

[26] Zhang C., Shi S., Liu Z., Yang F., Yin G. (2019). Drought tolerance in alfalfa (*Medicago sativa* L.) varieties is associated with enhanced antioxidative protection and declined lipid peroxidation. J. Plant Physiol..

[27] Iwuala E., Odjegba V., Sharma V., Alam A. (2020). Drought stress modulates expression of aquaporin gene and photosynthetic efficiency in Pennisetum glaucum (L.) R. Br. genotypes. Curr. Plant Biol..

[28] Chen J.H., Chen S.T., He N.Y., Wang Q.L., Zhao Y., Gao W., Guo F.Q. (2020). Nuclear-encoded synthesis of the D1 subunit of photosystem II increases photosynthetic efficiency and crop yield. Nat. Plants.

[29] Zhang Y.N., Zhuang Y., Wang X.G., Wang X.D. (2024). Evaluation of growth, physiological response, and drought resistance of different flue-cured tobacco varieties under drought stress. Front. Plant Sci..

[30] Ahmad M.A., Javed R., Adeel M., Rizwan M., Yang Y. (2020). PEG 6000-stimulated drought stress improves the attributes of in vitro growth, steviol glycosides production, and antioxidant activities in *Stevia rebaudiana* Bertoni. Plants.

[31] Fan H.F., Ding L., Xu Y.L., Du C.X. (2017). Antioxidant system and photosynthetic characteristics responses to short-term PEG-induced drought stress in cucumber seedling leaves. Russ. J. Plant Physiol..

[32] Bondok A.E.T., Mousa W.M., Rady A.M., Saad-Allah K.M. (2022). Phenotypical, physiological and molecular assessment of drought tolerance of five Egyptian teosinte genotypes. J. Plant Interact..

[33] Zhang A., Liu M., Gu W., Chen Z., Gu Y., Pei L., Tian R. (2021). Effect of drought on photosynthesis, total antioxidant capacity, bioactive component accumulation, and the transcriptome of *Atractylodes lancea*. BMC Plant Biol..

[34] Thayale Purayil F., Rajashekar B.S., Kurup S., Cheruth A.J., Subramaniam S., Hassan Tawfik N., MA Amiri K. (2020). Transcriptome profiling of *Haloxylon persicum* (Bunge ex Boiss and Buhse) an endangered plant species under PEG-induced drought stress. Genes.

[35] Feng J., Wang L., Wu Y., Luo Q., Zhang Y., Qiu D., He G. (2019). TaSnRK2. 9, a sucrose non-fermenting 1-related protein kinase gene, positively regulates plant response to drought and salt stress in transgenic tobacco. Front. Plant Sci..

